# Down Syndrome Related Muscle Hypotonia: Association with *COL6A3* Functional SNP rs2270669

**DOI:** 10.3389/fgene.2013.00057

**Published:** 2013-04-22

**Authors:** Arpita Dey, Krishnendu Bhowmik, Arpita Chatterjee, Pit Baran Chakrabarty, Swagata Sinha, Kanchan Mukhopadhyay

**Affiliations:** ^1^Manovikas Biomedical Research and Diagnostic CentreKolkata, West Bengal, India; ^2^Anatomy Department, Calcutta Medical CollegeKolkata, West Bengal, India

**Keywords:** Down syndrome, muscle hypotonia, congenital heart disease, functional SNP, *COL6A3*

## Abstract

Down syndrome (DS), the principal cause for intellectual disability, is also associated with hormonal, immunological, and gastrointestinal abnormalities. Muscle hypotonia (MH) and congenital heart diseases (CHD) are also frequently observed. Collagen molecules are essential components for maintaining muscle integrity and are formed by the assembly of three chains, alpha 1–3. The type VI collagen is crucial for cardiac as well as skeletal muscles. The COL α1 (VI) and α2 (VI) chains are encoded by genes located at the 21st chromosome and are expected to have higher dosage in individuals with DS. The α 3 (VI) chain is encoded by the *COL6A3* located at the chromosome 2. We hypothesized that apart from *COL6A1* and *COL6A2*, *COL6A3* may also have some role in the MH of subjects with DS. To find out the relevance of *COL6A3* in DS associated MH and CHD, we genotyped two SNPs in *COL6A3*, rs2270669 and rs2270668, in individuals with DS. Subjects with DS were recruited based on the Diagnostic and Statistical Manual for Mental Disorders-IV and having trisomy of the 21st chromosome. Parents of individuals with DS and ethnically matched controls were enrolled for comparison. Informed written consent was obtained for participation. Peripheral blood was used for isolation of genomic DNA. Target genetic loci were studied by DNA sequence analysis. Data obtained was subjected to population – as well as family-based statistical analysis. rs2270668 was found to be non-polymorphic in the studied population. rs2270669 showed significant association of the “C” allele and “CC” genotype with DS probands having MH (*P* = 0.02). Computational analysis showed that rs2270669 may induce structural and functional alterations in the COL α3 (VI). Interaction of COLα3 (VI) with different proteins, crucial for muscle integrity, was also noticed by computational methods. This pioneering study on *COL6A3* with DS related MH thus indicates that rs2270669 “C” could be considered as a risk factor for DS related MH.

## Introduction

Down syndrome (DS), the most common genetic cause for intellectual disability is characterized by the presence of one extra copy of the human chromosome 21 (Hsa21) (Roizen and Patterson, 2003; Rachidi and Lopes, 2007). The commonly accepted hypothesis to correlate the extra Hsa21 with DS pathophysiology is overexpression of genes present in the Hsa21 (Chou et al., 2008). However, changes in the expression of other euploid genes, possibly due to the presence of an extra Hsa21, were also speculated to contribute to the phenotypic variations in DS (Chou et al., 2008).

Down syndrome, with a great variability in the penetrance level, is characterized by complex phenotypic features, including morphological abnormalities of head and limbs, short stature, hypotonia, and hyperlaxity of the ligament etc. Malfunction of organs, particularly the heart (50% of newborns with DS), gastrointestinal tract obstructions or dysfunctions (4–5% of newborns with DS), increased risk of leukemia, early onset of Alzheimer like neuropathology are also common in individuals with DS (Antonarakis and Epstein, 2006; Rachidi and Lopes, 2007). Almost all children with DS suffer from muscle hypotonia (MH), a state of reduced muscle tone, usually related to the skeletal muscles. Due to MH, delay in developmental milestones, mastication problems (due to poor neuromuscular control), muscular weakness and dental anomalies are also of common occurrence in DS (Faulks et al., 2008). Besides skeletal muscle abnormality, congenital heart disease (CHD) due to cardiac muscle malformation is also a frequent problem (Klewer et al., 1998; Gittenberger-de Groot et al., 2003). Nearly 70% of atrio-ventricular (AV) canal defects are diagnosed in infants with DS (Korenberg et al., 1992).

Collagen, the trimeric extracellular matrix protein, is an important component for the formation of skeletal as well as cardiac muscles (Gordon and Hahn, 2010). It is the most abundant protein in mammals and is found in large quantities in the tendon, ligament, fascia, and skin (Bader et al., 2009). Collagen forms 1–2% of muscle tissue and 6% of strong muscles with tendons (Sikorski and Zdzislaw, 2001). As a component of the extracellular matrix, it is also present in bone, cartilage, inter-vertebral disk, cornea, lens, blood vessels, and intestine (Gordon and Hahn, 2010). Collagen is comprised of three helices, α1, α2, α3, which differ from each other in the amino acid compositions (Eastoe, 1967). Thirty different types of collagens based on the supra-molecular assembly and functional properties were reported (Kumar et al., 2007).

Among different types of collagen, the type VI (COLVI) has a crucial role in the function and stability of skeletal (Sabatelli et al., 2001) and cardiac (Klewer et al., 1998) muscles. Assembly of COLVI is a complex multistep process; equimolar quantities of three genetically distinct subunits, α1(VI), α2(VI), and α3(VI), associate to form a triple helical monomer which is then bonded in an anti-parallel direction to form dimers. These dimers align to form tetramers with the help of disulfide linkage (Furthmayr et al., 1983). In humans, genes encoding for the α1 and α2 chain of type VI collagen (*COL6A1* and *COL6A2* respectively) are located on the long arm of Hsa21, 21q22.3, a region determined to be critical for CHD associated with trisomy 21 (Francomano et al., 1991). Investigators have shown that mutations in three COL6 genes result in Bethlem myopathy (BM), Ulrich congenital muscular dystrophy (UCMD) (Baker et al., 2005; Lampe and Bushby, 2005), and CHD especially in the region of AV canal (Gittenberger-de Groot et al., 2003).

The α3 (VI) chain has a larger molecular mass than the α1 (VI) and α2 (VI) due to additional amino acids in the carboxy-terminal end (Bonaldo and Colombatti, 1989; Chu et al., 1990). Further, size of the α3 (VI) chain can vary from 2970 to 3176 amino acids due to alternative splicing of two exons (Stokes et al., 1991) and differential initiation of transcription (Zanussi et al., 1992). It is encoded by the *COL6A3* gene (ID 1293) containing 44 exons (43 coding) and is located on the chromosome 2 (2q37.3), proximal to the fibronectin locus (Weil et al., 1988). Being an important part of COLVI, abnormal transcription of *COL6A3* may hamper function of the protein, thus disrupting co-ordinated regulation of the collagen tetramer. Association of microdeletion at 2q37 with different physical abnormalities was first presented in 1990s (Oley et al., 1993; Wilson et al., 1995). Investigators have also reported deletion of 2q37 in cases exhibiting CHD/MH (Rauch et al., 1996).

A number of SNPs in the *COL6A3* have been investigated by different investigators for association with BM and UCMD; significant deleterious effect was predicted for some of these SNPs (Lamande et al., 1999, 2006; Demir et al., 2002; Baker et al., 2005; Baker and Rowland, 2007). However, till date, no investigation on *COL6A3* has been carried out in DS patients. We hypothesized that apart from *COL6A1* and *COL6A2*, expected to have an altered dosage due to trisomy of Hsa21, *COL6A3* may also have a role in DS associated MH and CHD. In order to test the hypothesis, in this preliminary investigation we studied two missense coding SNPs in the 41st exon of *COL6A3*, rs2270668 (A/G) and rs2270669 (C/G), in families with DS probands and compared the data with ethnically matched controls. rs2270668 (NC000002.11:g.238243481 A > G) and rs2270669 (NC000002.11:g.238243464 C > G) are located in the α3(VI) C4 domain, which has a minor role in the microfibril assembly (Lamande et al., 2006). Since these SNPs were never reported to have any major contribution in any disorder, we looked for their possible contribution in DS.

## Materials and Methods

### Subject recruitment

Unrelated nuclear families (*N* = 174) with DS probands [97 complete parent-offspring trios, 63 duos (54 without father and 9 without mother) and 14 single proband with DS] were recruited from the Out patient Department of Manovikas Kendra, Kolkata and Department of Paediatrics, Calcutta Medical College, Kolkata, based on the Diagnostic and Statistical Manual of Mental Disorders-IV (American Psychiatric Association, 1994). Age range of the probands was 8 months to 27 years (Mean ± SE 7.7 ± 0.51). All the DS cases recruited were confirmed for trisomy of the 21st chromosome by karyotyping. Signs of MH and CHD were assessed in probands by clinicians based on published literature (Morris et al., 1982; Spicer, 1984). Ethnically matched healthy control individuals (*N* = 205, mean age 8.9 ± 0.7) without any clinical history of intellectual disability, muscle and heart abnormality were also recruited. All the individuals were engaged for the study after obtaining informed written consent for participation. The study protocol was approved by the Institutional Human Ethical Committee.

### Genomic DNA isolation and genotyping

Peripheral blood samples were collected from the participants and genomic DNA was isolated using standard techniques (Miller et al., 1988).

A 410 bp sequence of the *COL6A3* gene, including the two selected SNPs, was amplified by polymerase chain reaction using forward primer 5′ ATTTCCTCTCTCGCTCATGC 3′ and reverse primer 5′ TGTCTCCTTTGTGTCCTATTTGA 3′. Amplicons generated were analyzed for restriction fragment length polymorphism of rs2270668 and rs2270669 with *Hin*fI and *Hae*III restriction enzymes respectively (Table 1).

**Table 1:** **Details of restriction fragment length polymorphism analysis**.

SNP IDs	Alleles	RE used	Cut site of RE^a^	Genotype	Length of fragments (bp)	Reaction condition
rs2270668	A/G	*Hin*fI	5′ **G** 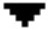 ANTC 3′ 3′ CTNA 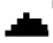 ****G**** 5′	AA AG GG	410 410/283/127 283/127	5 μl Amplicon incubated overnight at 37°C with 1× Genei buffer C and 1 U *Hin*fI in 20 μl reaction mixture, resolved in 2.5% agarose gel
rs2270669	C/G	*Hae*III	5′ GG 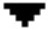 **C**C 3′ 3′C**C** 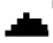 GG 5′	GG GC CC	250/160 250/160/143/107 160/143/107	5 μl Amplicon incubated overnight at 37°C with 1× Genei buffer C and 1 U *Hae*III in 20 μl reaction mixture, resolved in 15% polyacrylamide gel

*^a^Bold and underlined bases are the SNP position and the black arrows are the cutting position of the restriction enzyme (RE)*.

### Statistical analysis of genotype data

Simple r × c contingency table^1^ was used for testing the allelic and genotypic frequencies. Minor allele frequency in the eastern Indian control population (IND) was compared with four populations studied in the HapMap; namely, Caucasians from Utah with ancestry from western and northern Europe (CEU), Han Chinese from Beijing, China (HCB), Japanese from Tokyo, Japan (JPT), and Yoruba from Ibadan, Nigeria (YRI). Odds ratio calculator^2^ was used for measuring allelic odds ratios. Allelic transmission from parent to probands was analyzed by the Transmission Disequilibrium Test (TDT) program of Unphased (Version 2.403) (Dudbridge, 2003). By the TDT (Spielman et al., 1993) allelic transmission from heterozygous parents to affected offspring can be calculated. Power of all the chi square tests was calculated by Piface (Lenth, 2007).

### *In silico* analysis

Risk conferred by rs2270669 was analyzed computationally by FastSNP^3^ and F-SNP^4^. Structural change of the protein due to non-synonymous amino acid substitution was analyzed by the Globplot 2.3^5^. Interaction between COL α3 (VI) and other proteins was analyzed using String^6^. Expressional correlation of *COL6A3* with other genes involved in muscle development was analyzed by BioGPS.

## Results

rs2270668 was found to be monomorphic for the “A” allele in the studied eastern Indian population (*N* = 189) and no further analysis was carried out for this SNP.

rs2270669 was polymorphic and data obtained for this site was subjected to population – as well as family-based analysis. Genotype frequency was in Hardy–Weinberg equilibrium for all the groups. Comparison of allelic and genotypic frequencies revealed significant difference for the studied IND population as compared to the CEU and JPT (Table 2), principally due to an increase in the “G” allele with a concomitant decrease in the “C” allele in the IND population. Comparison with HCB also revealed a trend toward increase in the “G” allele frequency in the IND population along with a significant difference in genotypic frequencies (Table 2). As appeared from the HapMap data, in the YRI population this site was monomorphic for the “C” allele.

**Table 2:** **Comparative analysis of allelic and genotypic frequencies in different populations**.

Populations	Allelic frequency		χ^2^, *p* value	Genotypic frequency			χ^2^, *p* value
	C	G		CC	GC	GG	
CEU	0.793	0.207	**4.86, 0.027**	0.603	0.379	0.017	**11.1, 0.004**
HCB	0.756	0.244	2.91, 0.088	0.600	0.311	0.089	**6.53, 0.038**
JPT	0.784	0.216	**4.15, 0.042**	0.591	0.386	0.023	**10.6, 0.005**
YRI	1.000	0.000	**42.4, 0.000**	1.000	0.000	0.000	**81.7, 0.000**
IND	0.649	0.351	–	0.419	0.459	0.122	–

*CEU, Caucasians from Utah with ancestry from western and northern Europe; HCB, Han Chinese from Beijing, China; JPT, Japanese from Tokyo, Japan; YRI, Yoruba from Ibadan, Nigeria; IND, Eastern Indian control population*.

*Significant differences are mentioned in bold*.

Case-control association analysis revealed an increase in the “C” allele frequency in probands with DS as compared to eastern Indian control subjects; however, the difference was statistically insignificant (Table 3). Analysis of subjects sorted on the basis of gender failed to reveal any significant difference for male probands while female probands showed a trend toward increase in the “C” allele and “CC” genotype (Table 3). Comparative analysis of probands with DS sub-grouped on the basis of phenotypic characteristics revealed significant increase in the occurrence of the “C” allele (χ^2^ = 4.86, *p* value = 0.027) as well as “CC” genotype (χ^2^ = 10.7, *p* value = 0.005) in probands with DS having MH (*N* = 29) as compared to the controls. However, there was no significant difference in allelic or genotypic frequencies in probands with DS having CHD (*N* = 22). Probands with DS having MH and/or CHD (*N* = 47) also showed significantly higher occurrence of the “CC” genotype with concomitantly low GC/GG genotypes in comparison to controls (χ^2^ = 7.32, *p* value = 0.026).

**Table 3:** **Allelic and genotypic frequencies observed in different groups**.

Types	Study group	Allelic frequency		χ^2^, *p* value	OR (CI)	Genotypic frequency			χ^2^, *p* value
		C	G			CC	GC	GG	
All subjects	Control (*N* = 205)	0.649	0.351	–	–	0.419	0.459	0.122	–
	Father (*N* = 106)	0.708	0.292	0.827, 0.363	1.318 (0.73–2.39)	0.462	0.491	0.047	3.16, 0.206
	Mother (*N* = 151)	0.688	0.312	0.362, 0.547	1.199 (0.66–2.16)	0.450	0.477	0.074	1.46, 0.481
	Proband (*N* = 174)	0.724	0.276	1.14, 0.287	1.38 (0.76–2.52)	0.512	0.425	0.063	2.97, 0.226
Subjects categorized based on sex	Male Control (*N* = 70)	0.693	0.307	–	–	0.5	0.386	0.114	–
	Male Proband (*N* = 89)	0.708	0.292	0.952E–01, 0.758	1.1 (0.60–2.01)	0.483	0.449	0.067	1.36, 0.507
	Female Control (*N* = 121)	0.649	0.351	–	–	0.397	0.504	0.099	–
	Female Proband (*N* = 61)	0.754	0.246	2.38, 0.123	1.62 (0.88–2.98)	0.557	0.393	0.050	5.69, 0.058
DS with MH and/or CHD as compared to control	Proband with CHD (*N* = 22)	0.727	0.273	1.50, 0.221	1.46 (0.80–2.66)	0.500	0.455	0.045	4.70, 0.096
	Proband with MH (*N* = 29)	0.793	0.207	**4.86, 0.027**	2.03 (1.08–3.81)	0.621	0.345	0.034	**10.7, 0.005**
	Proband with CHD and/or MH (*N* = 47)	0.766	0.234	3.50, 0.061	1.80 (0.97–3.35)	0.574	0.383	0.043	**7.32, 0.026**
	Control (*N* = 205)	0.649	0.351	–	–	0.419	0.459	0.122	–

*OR, odds ratio; CI, 95% confidence interval; MH, muscle hypotonicity; CHD, congenital heart disease; DS, Down syndrome*.

*Significant differences are mentioned in bold*.

Family-based analysis by TDTphase failed to show any bias in transmission of any allele from the parents (from father/mother/both the parents) to the DS probands, irrespective of the gender of the proband (Table 4). Further analysis of DS probands having MH and/or CHD also failed to exhibit any bias in transmission of any allele from the parents (Table 4).

**Table 4:** **Frequency of rs2270669 allele transmission in families with DS probands**.

Sex	Parent	Allele	DS probands			DS probands with CHD and/or MH		
			Transmitted	Not-transmitted	χ^2^, *p* value	Transmitted	Not-transmitted	χ^2^, *p* value
All DS probands	Both	C	0.479	0.521	0.167, 0.683	0.523	0.476	0.048, 0.827
		G	0.521	0.479		0.476	0.523	
	Father	C	0.515	0.485	0.030, 0.862	0.500	0.500	0.000, 1.000
		G	0.485	0.515		0.500	0.500	
	Mother	C	0.424	0.576	0.761, 0.383	0.556	0.444	0.111, 0.739
		G	0.576	0.424		0.444	0.556	
Only male probands	Both	C	0.500	0.500	0.000, 1.000	0.429	0.571	0.143, 0.705
		G	0.500	0.500		0.571	0.429	
	Father	C	0.625	0.375	1.011, 0.315	0.500	0.500	0.000, 1.000
		G	0.375	0.625		0.500	0.500	
	Mother	C	0.400	0.600	0.805, 0.369	0.333	0.667	0.340, 0.560
		G	0.600	0.400		0.667	0.333	
Only female probands	Both	C	0.500	0.500	0.000, 1.000	0.571	0.429	0.287, 0.592
		G	0.500	0.500		0.429	0.571	
	Father	C	0.429	0.571	0.287, 0.592	0.500	0.500	0.000, 1.000
		G	0.571	0.429		0.500	0.500	
	Mother	C	0.600	0.400	0.403, 0.526	0.667	0.333	0.680, 0.410
		G	0.400	0.600		0.333	0.667	

*MH, muscle hypotonicity; CHD, congenital heart disease*.

Functional assessment of rs2270669 by SNPeffect revealed that it may be responsible for changing solvent accessibility of the protein. A potential change in splicing regulation by SC35 (in presence of C) and SF2 (in presence of G) was also observed. *In silico* analysis using String revealed that a number of proteins, important for muscle development as evidenced from Panther pathway tool^7^, can interact with COLα3 (VI) (Table 5).

**Table 5:** **Biological function of proteins having interaction with α3 (VI)**.

Proteins interacting with COL α3 (VI)	Biological processes involved
COL6A1	Macrophage activation, blood circulation, intracellular protein transport, receptor-mediated endocytosis, signal transduction, cell–cell adhesion, cellular component morphogenesis, ectoderm development, mesoderm development, skeletal system development, regulation of liquid surface tension, defense response to bacterium, asymmetric protein localization
LAMA2	Immune system process, muscle contraction, neurological system process, intracellular protein transport, endocytosis, signal transduction, cell–cell signaling, cell-matrix adhesion, protein modification process, cell motion, ectoderm development, mesoderm development, nervous system development, muscle organ development
ITGA7	Cell adhesion
COL1A2	Macrophage activation, blood circulation, intracellular protein transport, receptor-mediated endocytosis, signal transduction, cell–cell adhesion, cellular component morphogenesis, skeletal system development, angiogenesis, regulation of liquid surface tension, defense response of bacterium, asymmetric protein localization
GP6	Natural killer cell activation, cell surface receptor linked signal transduction, cell–cell signaling, blood coagulation
SDC1	Macrophage activation, signal transduction, cell adhesion, mesoderm development, skeletal system development
ITGA1	Cell adhesion
CD44	Immune system process, cell communication, cell adhesion
ITGA2B	Cell adhesion

Analysis by BioGPS revealed significant expressional correlation of *COL6A3* with *COL6A1*, *COL6A2*, *COL12A1*, *N1D1*, *MMP2*, and *MFAP5* (Table 6).

**Table 6:** **Genes showing expressional correlation with *COL6A3***.

Gene	Correlation coefficient
*COL6A1*	0.9071
*COL6A2*	0.8835
*NID1*	0.8377
*MMP2*	0.8291
*MFAP5*	0.8139
*COL12A1*	0.8107
*MFAP5*	0.8090

## Discussion

The α3 (VI) chain is a crucial component for skeletal as well as cardiac muscles. Terminal deletion in chromosome 2q harboring the *COL6A3* has been reported to lead to various disease phenotypes (Oley et al., 1993; Wilson et al., 1995; Rauch et al., 1996). DS related AV canal defect was reported to be associated with abnormalities in the COLVI and thus the molecule was speculated as an important candidate to study cardiac muscle development also (Davies et al., 1995; Baptista et al., 2000). Muscle related disorders like BM were reported to be associated with mutation in exon numbers 4, 5, 6, 7, 40, etc. of the *COL6A3* (Pepe et al., 1999; Lampe et al., 2005). Mutation in this gene was also reported to be associated with UCMD (Demir et al., 2002). A “A > G” transition in the splice-donor site of intron 29 was reported to cause deletion of the exon 29 (Demir et al., 2002). A nonsense mutation in the exon 5 (R465X), resulting in a shorter N-terminal domain of COLα3 (VI), was also reported. Another nonsense mutation (R2342X), leading to absence of collagen VI in muscle and fibroblasts, was reported to induce a severe phenotype of UCMD (Demir et al., 2002). However, investigation on rs2270669 in five UCMD patients revealed presence of the derived allele in heterozygous condition in only one individual; control individuals also showed presence of the SNP and it was inferred that this site may not have significant contribution in the disease etiology (Baker et al., 2005; Baker and Rowland, 2007). The current investigation is the first genetic association study on *COL6A3* rs2270668 and rs2270669 in subjects with DS.

rs2270668 (A/G) is responsible for a non-synonymous amino acid change [Lys (A) to Arg (G)] at amino acid position 3006 (codon 2) in the α3 (VI) chain. In eastern Indian subjects (*N* = 189), rs2270668 was non-polymorphic for the “A” allele. In other populations studied in the 1000 genome project (*N* = 208–218)^8^, MAF of this SNP was found to be only 0.0041. It may be inferred from the present study that this SNP does not have any significant role in the disease etiology.

rs2270669 (C/G) is located at amino acid position 3012 (codon 1) and is responsible for a missense amino acid change from proline (C) to alanine (G) in α3 (VI). *In silico* functional assessment of rs2270669 by SNPeffect revealed that it may be responsible for changing solvent accessibility of the protein. Substitution of residues in solvent accessible surface of a protein was reported to cause change in surface area affecting the backbone torsion potential (Gilis and Rooman, 1996). Our study also revealed a potential change in splicing regulation by SC35 (in presence of C) and SF2 (in presence of G). Analysis by GlobPlot 2.3 revealed that amino acid substitution (at 3012 amino acid position) may shorten length of the last globular domain in the α3 (VI) chain; in presence of alanine (“G” allele) the last globular domain may be formed from 2890 to 3072 amino acid region, while this domain comprises of only from 2890 to 3007 amino acid region in presence of proline (“C” allele). The disordered region predicted by the GlobPlot 2.3, was also changed from 3011–3021 to 3008–3021 amino acid in presence of alanine. Therefore, the non-synonymous change in rs2270669 could be crucial for proper structure and function of the α3 (VI) chain. However, the predicted functional effects are low to medium and thus may not cause significant amount of change. Moreover, the predictions have been made based on computational models and warrants further investigation for experimental validation.

Minor allele frequency of rs2270669 in the IND population showed severe dissimilarity with CEU and JPT. A significant difference in genotype frequencies of the control population was also noticed with the HCB. On the other hand, eastern Indian subjects with DS showed an allelic distribution pattern similar to the HCB and JPT. Population of the Indian subcontinent is highly heterogeneous (Indian Genome Variation Consortium, 2005). Subjects recruited for the present study are Indo-Caucasoid population from of the eastern part of India and we have noticed stark dissimilarities in genotypes of Indian population belonging to different geographical regions (Bhaduri et al., 2007). The Indo-Caucasoid subjects principally descends from the Euro-Caucasoid population, while migrants from mongoloid and Afro-Negroid population are prevalent still in the north-eastern and southern regions of India respectively (Ghosh and Seshadri, 2005). Allele frequency of a given SNP may get diluted by population admixer or by natural selection pressure, thus changing significantly from the ancestral populations. Whether this is the case for rs2270669 or is a genuine characteristic feature is a matter of conjecture at the moment and needs further validation in extended number of samples.

Though the present investigation showed a mild increase in the “C” allele and “CC” genotype as well as a concomitant decrease in the “GG” genotype in DS probands as compared to the controls, the difference was statistically insignificant. No preferential allelic distribution was found when the probands were categorized on the basis of gender; only a slightly higher trend was noticed for the “CC” genotype in the female probands. TDT analysis also failed to show any bias in transmission of any allele to DS probands from their parents. We may conclude from the present data that probands with DS have a higher trend of occurrence of the rs2270669 “C” allele. Whether this increase in the “C” allele is conferring any risk needs further corroboration with large cohort of samples.

Muscle hypotonia and CHD are common phenotypes in DS (Morris et al., 1982; Spicer, 1984; Gittenberger-de Groot et al., 2003). Our analysis in a limited number of subjects revealed a highly significant increase in the “C” (χ^2^ = 4.86, *p* value = 0.027, Power = 82.5%) allele [with a high odds ratio (OR = 2.03, CI = 1.08–3.81)] and “CC” genotype (χ^2^ = 10.7, *p* value = 0.005, Power = 97.74%) in probands with DS having MH as compared to the controls. Among the DS probands with CHD, an increase in “C” allele and “CC” genotype was also noticed. Comparison of the probands having MH and/or CHD with control also revealed a genotypic association (with increased “CC” and decreased “CG” and “GG” genotype; χ^2^ = 7.32, *p* value = 0.026, Power = 70.51%). Whether the “CC” genotype is conferring any advantage to probands with DS having MH/CHD warrants further investigation in the field.

Expression analysis showed that *COL6A3* has higher expressional correlation with *COL6A1*, *COL6A2*, *COL12A1*, *N1D1*, *MMP2*, and *MFAP5. In silico* interaction analysis also showed significant interaction of COLα3 (VI) with COL61, LAMA2, ITGA7, SDC1, etc. COL6A1 is involved in mesoderm development, skeletal muscle development, cell–cell adhesion etc. and may play an important role in cardiac and skeletal muscle development. COL1A2 and SDC1 also have roles in mesoderm and skeletal muscle development while LAMA2 regulates muscle contraction and muscle organ development. COL6A1, COL6A2, LAMA2, and SDC1 are also essential for cardiac functioning as they are involved in events like mesoderm development and angiogenesis. Thus COLα3 (VI) along with these proteins may have important attribution in CHD and MH. Few other genes, viz. DMD, HLA-A, HLA-B, HLA-DRB1, SPP1, PABPN1, COL6A1, DUX4, CAPN3, FCMD, ATXN3, retrieved from the Genetic disease association database^9^, may also have role in the development of muscle disorders.

*In silico* analysis in the present investigation revealed that allelic substitution of rs2270669 may alter globular domain formation, solvent accessibility, and splicing of the protein. We have also noticed an increase in the “C” allele in the DS probands, especially in those with MH/CHD. This may lead to an alteration in function of the COLα3 (VI) thus modifying the coordinate functional tetrameric structure of COLVI and play a vital role in DS related MH/muscle dysfunction. Major drawback of the current study is the limited number of DS probands with MH/or CHD. Additionally, hypothesis based on computational methods need to be validated by experimental evidences. To understand the actual role played by *COL6A3*, or in other words rs2270669, in the etiology of DS related MH further investigation is warranted in an extended cohort of subjects with DS.

## Conflict of Interest Statement

The authors declare that the research was conducted in the absence of any commercial or financial relationships that could be construed as a potential conflict of interest.

## Footnotes

^1^http://www.physics.csbsju.edu/stats/contingency_NROW_NCOLUMN_form.html

^2^http://www.hutchon.net/ConfidORnulhypo.htm

^3^http://fastsnp.ibms.sinica.edu.tw

^4^http://compbio.cs.queensu.ca/F-SNP/

^5^http://globplot.embl.de/

^6^http://string-db.org/

^7^http://www.pantherdb.org/pathway/

^8^http://www.ncbi.nlm.nih.gov/SNP/snp_ref.cgi?rs=rs2270668

^9^http://geneticassociationdb.nih.gov/

## Abbreviations

AV, atrio-ventricular; BM, Bethlem myopathy; CEU, Caucasians from Utah with ancestry from western and northern Europe; CHD, congenital heart disease; CI, 95% confidence interval; COLVI, collagen type VI; DNA, deoxy ribonucleic acid; DS, Down syndrome; HCB, Han Chinese from Beijing, China; Hsa21, Human chromosome 21; IND, Indian control population; JPT, Japanese from Tokyo, Japan; MH, muscle hypotonia; N, number of sample; OR, odds ratio; PCR, polymerase chain reaction; RE, restriction enzyme; SNP, single nucleotide polymorphism; UCMD, Ullrich congenital muscular dystrophy; YRI, Yoruba from Ibadan, Nigeria.
